# Peptide–Drug Conjugates for Targeted Delivery in Hematological Malignancies: From Design Principles to Clinical Application

**DOI:** 10.3390/pharmaceutics18070849

**Published:** 2026-07-13

**Authors:** Ningdan Zhou, Mengyuan Li, Yanyan Huang, Yanjun Wang, Jinghua Wang

**Affiliations:** 1 Department of Hematology, Guangdong Provincial People’s Hospital (Guangdong Academy of Medical Sciences), Southern Medical University, Guangzhou 510080, China; 2 School of Medicine, South China University of Technology, Guangzhou 510006, China; 3 Department of Urology, Sun Yat-sen University Cancer Center, Guangzhou 510060, China; 4 State Key Laboratory of Oncology in Southern China, Guangzhou 510060, China; 5 Guangdong Provincial Clinical Research Center for Cancer, Guangzhou 510060, China

**Keywords:** peptide–drug conjugate, antibody–drug conjugate, hematological malignancy, multiple myeloma, Melflufen

## Abstract

Hematological malignancies account for over 1.3 million new cases and approximately 700,000 deaths annually. Despite advances in targeted therapies, immunotherapies, and antibody–drug conjugates, relapse, refractory disease, and acquired drug resistance remain critical challenges. Peptide–drug conjugates (PDCs) have emerged as a promising targeted delivery platform, combining peptide-mediated specificity with potent cytotoxic payloads. In this review, we summarized the fundamental design principles of PDCs, including targeting peptide selection, linker engineering, and payload optimization, with emphasis on the biological characteristics of hematological malignancies. We then examined current preclinical and clinical progress across multiple myeloma, acute myeloid leukemia, myelodysplastic syndromes, B-cell non-Hodgkin lymphoma, and chronic myeloid leukemia. We further discussed emerging strategies such as cathepsin B-responsive PROTAC-PDC hybrids, nanotechnology-assisted delivery, and artificial intelligence-guided molecular design. Finally, we addressed key translational challenges, including tumor heterogeneity, payload resistance, and pharmacokinetic constraints, and proposed future directions toward biomarker-driven precision PDC therapy for hematological malignancies.

## 1. Introduction

Hematological malignancies are a diverse group of tumors affecting the blood, bone marrow, and lymphatic systems. According to the fifth edition of the World Health Organization (WHO) classification of hematolymphoid tumors (2022), these malignancies are broadly categorized into leukemia, lymphoma, and multiple myeloma (MM), each encompassing multiple subtypes with distinct genetic and clinical features [1,2]. Globally, hematologic malignancies represent a significant and growing disease burden. According to GLOBOCAN 2022 estimates, non-Hodgkin lymphoma (NHL) accounted for approximately 553,000 new cases and 251,000 deaths worldwide, with an age-standardized incidence rate (ASIR) of 5.6 per 100,000 population, making it the tenth most commonly diagnosed malignancy. Leukaemia contributed an additional 487,000 incident cases and 305,000 deaths (ASIR: 5.3 per 100,000), ranking as the tenth leading cause of cancer-related mortality. MM was responsible for 188,000 new cases and 121,000 deaths (ASIR: 1.8 per 100,000), while Hodgkin lymphoma, though less prevalent with 82,000 cases and 23,000 deaths (ASIR: 0.95 per 100,000), remains a major concern among adolescents and young adults. Collectively, these hematologic malignancies account for over 1.3 million new diagnoses and 700,000 deaths annually, underscoring the urgent need for more effective and better-tolerated therapeutic strategies [3]. The therapeutic landscape has been transformed over the past two decades by the introduction of multiple classes of targeted agents: proteasome inhibitors (PIs), immunomodulatory drugs (IMiDs), and anti-CD38 monoclonal antibodies for MM; BCL-2 inhibitor for acute myeloid leukemia (AML) and chronic lymphocytic leukemia; BCR-ABL tyrosine kinase inhibitors (TKIs) for CML and acute lymphocytic leukemia; chimeric antigen receptor T-cell (CAR-T) therapies for leukemia, lymphoma and MM; and antibody–drug conjugates (ADCs) such as brentuximab vedotin, polatuzumab vedotin, and belantamab mafodotin for lymphoma and MM [4,5,6]. Despite these advances, most patients with hematological malignancies eventually develop multi-drug resistant, relapsed/refractory disease, for which treatment options remain limited and prognosis is poor [5]. This persistent unmet need has driven the search for therapeutic platforms that can overcome the intrinsic limitations of both conventional chemotherapy and current biological agents.

ADCs have validated the concept of targeted cytotoxic delivery, with 21 agents approved worldwide by the end of 2025 [7,8,9]. However, ADCs are constrained by their large molecular size (~150 kDa), which limits tumor penetration, as well as by high manufacturing costs, batch-to-batch heterogeneity, and the potential for immunogenicity [7,10]. Peptide–drug conjugates (PDCs)—in which a tumor-homing or cell-penetrating peptide is covalently linked to a cytotoxic payload via a cleavable or non-cleavable linker—extend the ADCs paradigm to a smaller, more versatile format [7,10,11,12,13]. PDCs share a similar concept with ADCs, but have distinct structures and properties. PDCs offer several potential advantages over ADCs. Their small size enables deeper tissue penetration; their chemical synthesis ensures homogeneous products with lower manufacturing costs; their peptide backbone has lower immunogenicity; and their rapid renal clearance, while limiting half-life, reduces the risk of cumulative systemic toxicity [7,10,14,15]. Two PDCs have received regulatory approval: ^177^Lu-dotatate (Lutathera^®^) for neuroendocrine tumors, and melphalan flufenamide (Pepaxto^®^/Pepaxti^®^) for RRMM [11,16]. The latter, although subsequently withdrawn from the U.S. market after a confirmatory phase III trial, remains approved by the European Medicines Agency and offers critical insights into both the promise and the design challenges of PDCs in hematological malignancies [11].

In this review, we will first outline the structural components that govern PDC function, with an emphasis on linker chemistry and payload selection. Then we comprehensively survey the application of PDCs across the major hematological malignancies—MM, AML, myelodysplastic syndromes (MDS), B-cell non-Hodgkin lymphoma (B-NHL), and lymphoid leukemias—integrating preclinical evidence with the latest clinical data. Finally, we discuss key challenges and emerging strategies, including innovative linker design, PROTAC-PDC hybrids, and nanotechnology-enabled delivery, that are poised to shape the next generation of PDCs therapeutics for hematological malignancies.

## 2. Molecular Design Principles of PDCs

**Figure 1:**
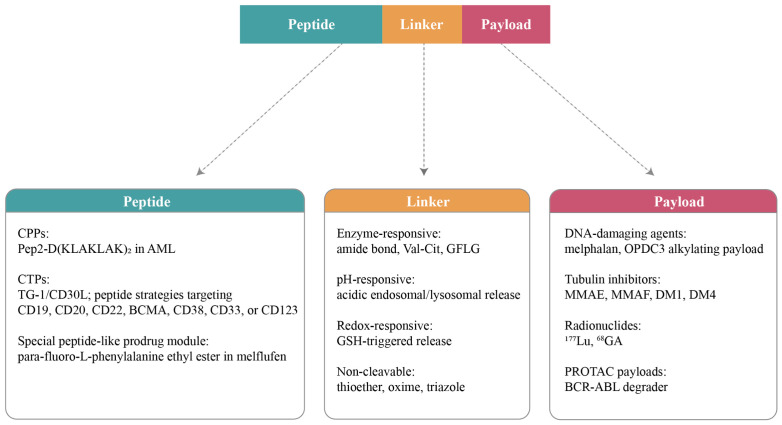
Modular architecture and classification framework of PDCs. Abbreviations: CD30L, CD30 ligand; BCMA, B-cell maturation antigen; GSH, glutathione; Val–Cit, valine–citrulline; GFLG, glycine-phenylalanine–leucine–glycine tetrapeptide; MMAE, monomethyl auristatin E; MMAF, monomethyl auristatin F; DM1, mertansine (maytansinoid DM1); DM4, ravtansine (maytansinoid DM4); PROTAC, proteolysis-targeting chimera; BCR-ABL, breakpoint cluster region–Abelson fusion protein.

PDCs are composed of three modular components: a tumor-homing peptide, a cytotoxic payload, and a linker. These three parts act in tandem to transport cytotoxic agents toward specific receptors expressed on cancer cells [10,17]. Each individual component contributes to the overall selectivity, pharmacokinetic profile, and therapeutic window of the conjugate [18]. Collectively, they form a modular design platform. Unlike traditional chemotherapeutics with fixed molecular structures, this system supports gradual structural optimization tailored to the distinct biological traits of different malignancies. A critical distinction between hematological malignancies and solid tumors lies in the drug delivery mechanisms available to each. In solid tumors, the enhanced permeability and retention (EPR) effect—arising from leaky tumor vasculature and impaired lymphatic drainage—enables the passive accumulation of macromolecular therapeutics, including ADCs, within the tumor interstitium [10,19]. However, the clinical relevance of the EPR effect in vivo remains limited. In a systematic analysis of 117 studies published between 2005 and 2015, Wilhelm and colleagues reported that the median accumulation of injected nanoparticles in solid tumors was only 0.7% in mouse tumor models, with no noticeable improvement over the study period [20]. Consistent with these findings, quantitative imaging studies by Sindhwani and colleagues showed that, in mouse tumor models, nanoparticle delivery to solid tumors relies primarily on active trans-endothelial transport rather than passive leakage. Inter-endothelial gaps accounted for only 3–25% of nanoparticle accumulation, depending on particle size (3% for 50 nm and 25% for 100 nm gold nanoparticles) [21]. Taken together, these studies indicate that nanocarrier-based formulations designed to rely on EPR-mediated passive accumulation may deliver much lower drug levels to tumors than previously expected. In contrast, ADCs depend less on the EPR effect because of antibody-mediated active targeting, although they still face the same biological barriers to tumor penetration and bone marrow accessibility. Unlike solid tumors, hematological malignancies do not form a vascularized tumor mass. Instead, leukemic and myeloma cells circulate in the bloodstream and home to the bone marrow niche [19], where the EPR effect is essentially absent. As a result, selective drug delivery cannot depend on passive accumulation but instead requires active receptor-mediated targeting [17,22]. This difference directly shapes the design of PDCs. Each of the three key components—the targeting peptide, linker, and payload—needs to be optimized for the circulatory system and bone marrow microenvironment, where target binding, linker stability, and intracellular payload release are governed by biological conditions distinct from those in solid tumors [10,19]. The following sections discuss the design considerations for each component, with a particular focus on their application in hematological malignancies. Figure 1 provides an overview of the three-component architecture of PDCs together with representative examples discussed in this review.

### 2.1. Three-Component Modular Architecture

The canonical PDC architecture resembles that of an ADC but is considerably smaller and chemically well defined. Beyond the advantages discussed in the Introduction, the three-component modular design offers an important engineering advantage: each building block can be independently optimized without redesigning the entire conjugate. The targeting peptide can be modified for receptor affinity or metabolic stability; the linker chemistry can be adapted to regulate release kinetics; and the cytotoxic payload can be selected against tumor-specific vulnerabilities [18]. The following sections discuss these three components in turn—homing peptides, linkers, and cytotoxic payloads—with particular focus on design principles relevant to hematological malignancies.

### 2.2. Homing Peptide

The homing peptide functions as the targeting component of a PDC, directing the cytotoxic payload to tumor cells or the tumor microenvironment. It largely determines the targeting selectivity, tumor penetration characteristics, in vivo stability, and tissue distribution profile of the entire conjugate [14]. The efficacy, pharmacokinetic/pharmacodynamic profile, and therapeutic index of a PDC are strongly influenced by peptide properties including affinity for the target receptor, resistance to serum proteases, and cell-penetrating ability. Unlike monoclonal antibodies (mAbs), peptides can serve not only as tumor-homing ligands for targeted delivery but also as cell-penetrating enhancers that facilitate the intracellular transport of small-molecule drugs [10]. According to their mechanisms of cellular entry, targeting peptides employed in PDCs can be broadly divided into two major categories: cell-penetrating peptides (CPPs) and cell-targeting peptides (CTPs). These two classes are discussed below in the order of their impact on PDC selectivity and efficacy [23].

#### 2.2.1. CPPs

The cell membrane is a major physiological barrier that limits the intracellular entry of macromolecules, proteins, nucleic acids, and many therapeutic agents [10]. Therefore, development of drug delivery strategies capable of crossing cancer cell membranes is critical for achieving effective intracellular destruction. Cell-penetrating peptides (CPPs) provide one such strategy by acting as versatile molecular transporters. These short peptides, typically ranging from 5 to 30 amino acid residues, are rich in arginine and lysine, endowing them with a net positive charge and amphiphilic properties under physiological conditions [10]. These properties allow CPPs to efficiently transport otherwise cell-impermeable compounds or drug payloads across the plasma membrane to their intracellular targets [10,23]. A preclinical example in hematological malignancies is Pep2-D(KLAKLAK)_2_, a CPP-based peptide–conjugate strategy developed for AML. In this construct, Pep2 functions as a TLR2-mediated cell-penetrating peptide, whereas D(KLAKLAK)_2_ serves as a proapoptotic effector peptide, enabling TLR2-dependent penetration into AML cells and followed by apoptosis [24].

The mechanisms by which CPPs penetrate the cell membrane are generally divided into two categories: direct translocation and endocytosis, although the relative contribution of each pathway can be influenced by the peptide structure and concentration of the peptide [10,23]. Direct translocation involves electrostatic interactions between positively charged CPPs and negatively charged membrane components, leading to membrane destabilization and transient pore formation [25]. Endocytic uptake, on the other hand, encompasses clathrin-mediated endocytosis and macropinocytosis, among other pathways [23]. From the perspective of PDC design, CPPs provide several advantages: (1) they can improve the solubility and reduce the non-specific toxicity of small-molecule drugs; (2) they can alter the cellular entry mechanism of a cytotoxic payload, thereby circumventing drug efflux pumps such as P-glycoprotein and overcoming multidrug resistance; and (3) they enable the intracellular delivery of therapeutics to tumors with low or heterogeneous expression of surface receptors [23,25].

#### 2.2.2. CTPs

Cell-targeting peptides (CTPs) recognize tumor cells through high-affinity binding to receptors or antigens that are overexpressed on the cell surface. This interaction triggers receptor-mediated endocytosis, allowing the PDC to be internalized into endosomes and subsequently trafficked to lysosomes for intracellular payload release [14,23,26]. This “lock-and-key” recognition mechanism gives CTP-based PDCs with a high degree of tumor selectivity. Several clinically validated cell-surface targets have been explored for CTP development in hematological malignancies. For example, B-cell malignancies commonly overexpress CD19, CD20, and CD22, which are widely used as targeting markers for B-lineage lymphomas and leukemias [22]. In MM, B-cell maturation antigen (BCMA) and CD38 have been clinically validated as ADC targets, and the corresponding targeting peptides are now being investigated for PDC development [27]. In myeloid leukemias, CD33 and CD123 (the IL-3 receptor α chain) are well-established surface targets for AML and MDS. Their clinical validation in ADCs also provides a strong basis for their application in PDCs [28]. More recently, additional targets have been identified for PDC development. For example, Shi et al. used whole-cell phage display technology to identify CD30 ligand (CD30L/TNFSF8) as a highly expressed surface marker across multiple B-NHL subtypes, including mantle cell lymphoma, Burkitt lymphoma, and diffuse large B-cell lymphoma, and subsequently discovered the high-affinity peptide TG-1. Notably, CD30L expression is minimal in normal B cells, suggesting a favorable therapeutic window [22]. A major limitation of CTPs is their reliance on stable, high-level expression of the target antigen on tumor cells. Antigen downregulation or loss is a well-recognized mechanism of therapeutic resistance in oncology, making target selection a key determinant of CTP-based PDC efficacy [7,10,23]. In addition, antigen heterogeneity within individual tumors (spatially and temporally) may limit PDCs binding of antigen-low subpopulations, resulting in incomplete tumor eradication [29]. Clonal evolution under therapeutic pressure can further enrich antigen-low or antigen-negative subclones, progressively reducing the tumor-selective targeting efficiency of CTP-based PDCs over the course of treatment [30]. Resistance mechanisms documented for ADCs—principally altered internalization, impaired lysosomal function, and drug efflux pump upregulation—may likewise reduce the efficacy of PDCs, because both modalities rely on receptor-mediated uptake and intracellular payload release [31]. Similarly, resistance mechanisms described for CAR-T therapy, such as alternative splicing that generates epitope-deficient isoforms and lineage switching, may also affect CTP-based PDCs directed against the same targets [32].

### 2.3. Linkers

The linker is the central structural element connecting the targeting peptide and the cytotoxic payload. It acts as a pharmacological switch that determines both the stability of the conjugate during systemic circulation and the release of the active drug at the intended site of action [17,33]. Linkers used in PDCs are broadly classified as either cleavable or non-cleavable.

Cleavable linkers take advantage of the distinct physiological and biochemical characteristics of tumors to trigger payload release and can be divided into three major categories: enzyme-responsive, pH-responsive, and redox-responsive linkers. Enzyme-responsive linkers are cleaved by proteases that are overexpressed in tumor cells. pH-responsive linkers utilize the acidic conditions of the tumor microenvironment (pH 6.5–6.9) and intracellular endosomes/lysosomes (pH 4.5–6.0), compared with the physiological pH of normal tissues (7.4), to induce drug release. Redox-responsive linkers respond to the marked intracellular reducing environment of tumor cells, where glutathione (GSH) concentrations are typically in the millimolar range, whereas plasma GSH levels remain in the low micromolar range [17,34].

Unlike cleavable linkers, non-cleavable linkers (e.g., thioether, oxime, and triazole bonds) undergo little or no chemical or enzymatic cleavage under physiological conditions. Instead, payload release depends on complete lysosomal degradation of the peptide backbone after endocytosis of the intact conjugate [10,34]. Non-cleavable linkers provide greater stability during circulation and therefore reduce off-target toxicity. However, the released payload generally retains linker- or amino acid-derived residues, which may influence intracellular drug activity and the bystander effect [17,34].

The choice of linker has a direct impact on the pharmacokinetic profile, therapeutic index, and safety of a PDC. Linker design should take into account the properties of the targeting peptide, the chemical characteristics of the payload, the biology of the target, and the therapeutic requirements of the intended indication. More recently, linker engineering has expanded toward architectures that respond to multiple tumor-associated signals, including enzymatic activity, acidic pH, redox conditions, or combinations of these stimuli, with the aim of improving tumor selectivity while reducing off-target toxicity [11,35,36]. Several representative PDCs illustrate this trend. Melflufen uses a simple amide bond that remains stable in plasma but is rapidly hydrolyzed by intracellular aminopeptidases overexpressed in myeloma cells, allowing selective intracellular release of melphalan while limiting systemic exposure to the free alkylating agent [37]. Similarly, BT5528, a bicyclic peptide–MMAE conjugate targeting EphA2, employs a valine–citrulline (Val-Cit) dipeptide linker that is cleaved by lysosomal cathepsin B, resulting in efficient intratumoral accumulation of MMAE with minimal circulating free payload [26]. This strategy has also been extended to emerging therapeutic modalities. For example, cathepsin B-cleavable GFLG tetrapeptide linkers have been incorporated into PROTAC-PDC constructs to enable selective intracellular release of BCR-ABL degraders in leukemia cells, highlighting the adaptability of enzyme-responsive linker design beyond conventional cytotoxic payloads [36].

Together, these examples show that linker technology has evolved from relatively simple single-stimulus-responsive systems toward more versatile and programmable designs. Incorporating multiple tumor-associated signals into linker design is expected to further improve the efficacy, selectivity, and safety of next-generation PDCs.

### 2.4. Cytotoxic Payload

Conjugation of a cytotoxic drug to a targeting peptide provides multiple pharmacological benefits: improved solubility, enhanced tumor selectivity, extended circulation time relative to the free drug, optimized bioavailability, and diminished off-target side effects and systemic toxicity [10,38]. An optimal cytotoxic payload is typically a low-molecular-weight compound that not only exhibits high potency and a well-characterized mechanism of action, but also retains its antitumor efficacy after chemical coupling to the peptide carrier [17]. The most common payload classes employed in PDC design include tubulin inhibitors, DNA damaging agents, and radionuclides [13].

Tubulin inhibitors act on microtubules with anti-mitotic abilities. Tubulin inhibitors disrupt the normal polymerization/depolymerization dynamics of tubulin, arresting tumor cells in the G_2_/M phase and driving them toward apoptosis. Vinca alkaloid analogs and paclitaxel (PTX) belong to this class of agents. Auristatins (e.g., monomethyl auristatin E [MMAE] and monomethyl auristatin F [MMAF]) and maytansinoids (e.g., DM1 and DM4) represent the two most extensively studied classes of tubulin-targeting payloads [17,39]. MMAE binds to the Vinca domain of tubulin and inhibits tubulin polymerization; it has been widely employed in clinically approved ADCs such as brentuximab vedotin and polatuzumab vedotin and is being progressively expanded into PDC development [39]. Similarly, the maytansinoid DM1 has been incorporated into several PDCs. PEN-221—a miniaturized drug conjugate (~2 kDa) comprising a Tyr^3^-octreotate peptide conjugated to DM1 via a cleavable disulfide linker—targets SSTR2 with high affinity (Ki = 51 pmol/L) and achieved complete tumor regressions in SSTR2-positive SCLC xenograft models at doses below the maximum tolerated dose, demonstrating deep tumor penetration and a large therapeutic window [40].

DNA damaging agents exert their antitumor activity through covalent modification or crosslinking of DNA, thereby blocking replication and transcription and triggering apoptosis [17]. Alkylating agents, anthracyclines, and topoisomerase inhibitors represent the most widely employed DNA-damaging payload classes in PDC design. Melflufen, an aminopeptidase-activated alkylating agent, exemplifies the alkylating class: its ethyl ester derivative of melphalan remains inert in plasma but is rapidly cleaved by intracellular aminopeptidases overexpressed in myeloma cells, releasing the active nitrogen mustard selectively within the tumor [27]. Anthracyclines such as doxorubicin (DOX) have also been extensively utilized as PDC payloads. For instance, a GnRH analog [D-Lys6]-GnRH conjugated to DOX demonstrated superior antiproliferative activity compared to free DOX in GnRH receptor-positive cancer models and progressed into clinical evaluation [10]. In addition, camptothecin and its derivatives (e.g., SN-38, exatecan)—topoisomerase I inhibitors that trap the topoisomerase I-DNA cleavage complex—have been employed in PDCs such as SAP-CPT, an RGD-targeted self-assembling peptide-camptothecin conjugate that enhanced antitumor efficacy in breast and bladder cancer xenografts [17], and CBX-12, a pHLIP-exatecan conjugate currently in Phase II clinical trials [41].

Radionuclides represent a versatile payload class in PDC design, functioning as either therapeutic or diagnostic agents depending on the isotope selected. Unlike conventional chemotherapy, radionuclide payloads deliver ionizing radiation directly to tumor cells through peptide-mediated receptor targeting, thereby maximizing tumor-localized cytotoxicity while sparing adjacent normal tissues [16,42]. The FDA-approved therapeutic PDC Lutathera (177Lu-DOTA-TATE) exemplifies this approach: its somatostatin analog Tyr^3^-octreotate binds SSTR2 with high affinity, the DOTA chelator stably coordinates the β-emitting radionuclide 177Lu, and upon receptor-mediated internalization, the retained intratumoral radiation induces DNA double-strand breaks within a short tissue penetration range (~2 mm), limiting off-target exposure [7]. A second FDA-approved radionuclide PDC, Pluvicto (177Lu-PSMA-617), employs a PSMA-targeting peptidomimetic with the same 177Lu-DOTA payload and received approval in 2022 for metastatic castration-resistant prostate cancer, further validating the radionuclide PDC platform [41]. In the diagnostic setting, 111In-DTPA-octreotide (Octreoscan) and 68Ga-DOTA-TATE demonstrate how an identical peptide-chelator scaffold can be radiolabeled with γ- or positron-emitting isotopes for tumor imaging and patient stratification, enabling a theranostic paradigm in which the same targeting framework serves both diagnosis and treatment [7]. Although these radionuclide PDCs are currently approved for neuroendocrine and prostate tumors rather than hematological indications, they are included here to illustrate the radionuclide payload class and to demonstrate that the underlying peptide-chelator design—receptor-mediated targeting coupled with a stably chelated therapeutic isotope—constitutes a transferable platform whose adaptation to hematological targets is actively being explored.

## 3. Melflufen—The First-in-Class PDC

In the evolution of PDCs from conceptual design to clinical translation, melphalan flufenamide (Melflufen, brand names: Pepaxto^®^/Pepaxti^®^) occupies an indispensable and pivotal position. Although the FDA withdrew approval of Melflufen in February 2024, the European Medicines Agency (EMA) and the United Kingdom’s Medicines and Healthcare products Regulatory Agency (MHRA) granted marketing authorization in August 2022 and November 2022, respectively, and it remains approved for use in both regions [5,43].

As the first chemotherapeutic PDC to receive FDA accelerated approval, it also represents the PDC with the richest clinical evidence base and the most instructive regulatory journey in hematological oncology so far [44]. From the elegant logic of its molecular design to the complex outcomes of its phase III confirmatory trial, Melflufen has served as an invaluable case study for the entire PDCs field—demonstrating the immense potential of PDCs to achieve tumor-selective drug delivery, while simultaneously exposing the fundamental challenges that remain to be addressed in target biology, payload sensitivity, and patient selection.

### 3.1. Structure of Melflufen

Melflufen is a typical PDC, which is an ethyl ester of a lipophilic dipeptide composed of melphalan and para-fluoro-L-phenylalanine. Owing to its high lipophilicity, it rapidly traverses the cell membrane and is subsequently hydrolyzed by intracellular aminopeptidases into the hydrophilic alkylating metabolites melphalan and desethyl-melflufen [45]. The core innovation of this structural design lies in the fact that the introduction of para-fluoro-L-phenylalanine ethyl ester endows the entire molecule with high lipophilicity, enabling it to rapidly traverse the lipid bilayer of the cell membrane via passive diffusion rather than depending on slow active transporters—such as L-type amino acid transporter 1 (LAT1), which is required for the cellular uptake of the conventional alkylating agent melphalan [46,47]. This transforms melphalan from a first-generation alkylating agent that relies on transporter-mediated uptake into a “molecular Trojan horse” capable of autonomous and efficient membrane translocation [35,46]. The cellular uptake efficiency of conventional melphalan is limited by the expression levels and functional status of transporters such as LAT1 on the tumor cell surface, which constitutes a major source of intrinsic drug resistance [27]. By bypassing this rate-limiting step, Melflufen fundamentally overcomes the poor cellular uptake of the parent drug melphalan [45,46,47].

### 3.2. Targeted Delivery and Intracellular Trapping Mechanism

The tumor selectivity of Melflufen is achieved through an elegant stepwise cascade mechanism—“passive diffusion–enzymatic activation–intracellular trapping”—which endows this PDC with its unique tumor-selective delivery capability.

#### 3.2.1. Passive Diffusion

Passive diffusion across the cell membrane. Owing to its high lipophilicity, Melflufen is capable of directly traversing the lipid bilayer of the cell membrane without relying on any transporters or receptors [35]. This property is particularly critical in the context of hematological malignancies—tumor cells within the bone marrow microenvironment are surrounded by dense stroma and hematopoietic cells, rendering large-molecule therapeutics such as ADCs difficult to penetrate effectively [47,48]. The small molecular size and high lipophilicity of Melflufen, by contrast, confer distinct tissue penetration advantages [27].

#### 3.2.2. Enzymatic Activation

The core of Melflufen’s design lies in its molecular disguise as a substrate for two classes of hydrolytic enzymes that are highly active in tumor cells. Once inside the cell, the ethyl ester group of Melflufen is first cleaved by esterases to generate the intermediate desethyl-Melflufen; this intermediate is then cleaved at the amide bond by aminopeptidases—including aminopeptidase N (APN/CD13), leukotriene A_4_ hydrolase (LTA4H), and leucine aminopeptidase 3 (LAP3)—to ultimately liberate the hydrophilic alkylating agents melphalan and desethyl-melflufen [49,50].

This enzymatic activation of Melflufen is not mediated by aminopeptidases alone, but is instead accomplished through the concerted action of both esterases and aminopeptidases. In a seminal experiment conducted by Wickström et al., treatment of tumor cells with the esterase-specific inhibitor ebelactone significantly reduced both the intracellular release of melphalan and the cytotoxicity of Melflufen—directly demonstrating that esterase-mediated hydrolysis of the ethyl ester bond is a prerequisite for the subsequent aminopeptidase-mediated cleavage of the amide bond [49].

Once inside the cell, Melflufen is rapidly consumed as it is converted by the two enzymes into the hydrophilic metabolites melphalan and desethyl-melflufen, causing a precipitous drop in the intracellular concentration of free Melflufen. This steepens the concentration gradient across the plasma membrane, which in turn drives the continued passive diffusion of additional extracellular Melflufen molecules into the cell—thereby establishing a self-amplifying positive-feedback cycle of “diffusion → hydrolysis → trapping → concentration gradient → further diffusion” [35]. As a result, the intracellular concentration of alkylating agents can reach levels that are several dozen times higher than those achievable with an equivalent dose of free melphalan, providing the molecular foundation for the subsequent induction of irreversible DNA damage and apoptosis [48].

Miettinen et al. analyzed MM patient samples and confirmed that several aminopeptidases are significantly overexpressed in myeloma cells compared with normal plasma cells, and that their expression levels are further upregulated with disease progression, providing molecular-level evidence to support the “tumor-selective activation” of Melflufen [50].

#### 3.2.3. Intracellular Trapping

Following enzymatic cleavage, the resulting metabolites melphalan and desethyl-melflufen undergo a sharp decline in logP, rendering them unable to passively diffuse back across the plasma membrane—a physicochemical transition that constitutes the mechanistic basis of the intracellular trapping effect described in Section 3.2.2. Quantitative analyses by Wickström et al. confirmed that the intracellular Cmax of melphalan liberated from Melflufen was at least tenfold higher than that achieved with equimolar free melphalan, with peak concentration reached within 15 min [49]. This trapping effect is strictly dependent on aminopeptidase activity: preincubation with bestatin significantly reduced intracellular melphalan accumulation, confirming that enzymatic cleavage is a prerequisite for drug retention [47].

### 3.3. Linker Chemistry and Payload Design

From the perspective of linker chemistry, Melflufen employs an amide bond to bridge its melphalan payload and the para-fluoro-L-phenylalanine ethyl ester targeting module. This minimalist architecture embodies deliberate and instructive rationale. As systematically reviewed by Alas et al., the amide bond occupies a distinctive position in the linker stability hierarchy—it is more resistant to spontaneous hydrolysis in plasma than ester or hydrazone linkers, yet remains susceptible to enzymatic cleavage by the aminopeptidases and esterases that are abundantly expressed in tumor cells [34]. In cell-free human plasma, Melflufen exhibits a half-life of approximately 5 h with negligible formation of free melphalan, demonstrating that the amide bond confers sufficient circulatory integrity to avoid premature payload release [47]. This stability profile positions Melflufen, from a linker chemistry perspective, as a paradigmatic example of the enzyme-cleavable linker design strategy: its amide bond linker remains intact in plasma yet becomes a highly efficient substrate for aminopeptidases and esterases upon entering tumor cells, with tumor-enriched proteases serving as the trigger for site-selective drug liberation [34].

The functional sophistication of Melflufen’s linker strategy, however, extends well beyond the selection of a single chemical bond. The true pharmacological activity resides not in the linker itself, but in the enzymatic activation step that the linker facilitates. The amide bond remains inert during systemic circulation, providing the prodrug with a stable protective window that prevents premature degradation; once inside the cell, it becomes the substrate for a sequential two-enzyme cleavage that is almost entirely confined to the intracellular compartment [47,49]. This design therefore satisfies the two cardinal requirements of PDC linker chemistry—plasma stability and tumor-selective release—not through an exotic chemical motif, but through a mechanism-based integration of linker lability with the biological context in which cleavage occurs [11,17].

A direct implication of this design logic is that the choice of payload must be matched not only to the disease indication but also to the mechanism of linker activation. In Melflufen, the payload is the alkylating agent melphalan, a drug whose own clinical limitations—poor tumor selectivity, transporter-dependent uptake, and the emergence of resistance in high-dose melphalan-pretreated patients—mirror the very problems that PDC technology seeks to overcome [27]. By converting melphalan from a transporter-dependent agent into a prodrug that enters cells via passive diffusion, Melflufen addresses the uptake bottleneck. Yet the clinical experience with Melflufen provides a cautionary illustration: optimizing intracellular delivery cannot fully compensate for pre-existing intrinsic resistance to the payload itself, as observed in patients with prior exposure to high-dose alkylating regimens [43,51].

The clinical experience with Melflufen thus crystallizes two fundamental design principles for PDCs in hematological malignancies. First, linker chemistry must be selected not merely for its chemical stability in isolation, but for its ability to couple tumor-specific enzymatic activation with prolonged circulatory integrity. This principle has informed the development of next-generation stimulus-responsive linkers. Cathepsin B-sensitive GFLG tetrapeptide spacers exploit the same principle of tumor-enriched protease activation but substitute aminopeptidases with cathepsin B as the enzymatic trigger, thereby expanding applicability to target-positive tumors with lower aminopeptidase expression. Dual-lock designs further incorporate two orthogonal cleavage requirements—such as low pH plus cathepsin B activity—to restrict payload release to the intracellular lysosomal compartment with even greater precision [11,17]. Second, the payload must be chosen with equal attention to its intrinsic resistance profile in the intended patient population: a PDC that merely “re-delivers” an old cytotoxin more efficiently may offer limited clinical benefit if the target population has already been exposed to—and developed resistance against—the same class of cytotoxic agent, regardless of how sophisticated its delivery mechanism may be [28,51]. These lessons, drawn from the most extensively studied PDC in the hematological setting, provide a conceptual framework for evaluating the next generation of PDCs currently under preclinical and clinical investigation for MM, AML, B-cell lymphomas, and lymphoid leukemias.

### 3.4. CMC and Formulation Design Implications

The design principles distilled from the linker chemistry and payload selection of Melflufen, as discussed above, must undergo rigorous chemistry, manufacturing, and controls (CMC) validation before clinical translation. Melflufen’s manufacturing experience offers broadly instructive insights in this regard.

First, single-site chemical conjugation fundamentally determines the homogeneity of a PDC. Melflufen is constructed via EDC/HOBt-mediated solution-phase amide Structural Advantages of the PDC Modality Over ADC coupling (89% yield, ≥99% purity) that directly links melphalan to para-fluoro-L-phenylalanine ethyl ester [52]. Its precisely defined drug-to-peptide ratio (DPR) of 1:1 is unattainable by ADCs, whose stochastic conjugation strategies inevitably generate batch-to-batch Drug-to-Antibody Ratio (DAR) heterogeneity and the resulting pharmacological variability [10].

Second, the analytical characterization framework of a small-molecule PDC is far simpler than that of an ADC—a direct consequence of chemical homogeneity at the quality control level. Melflufen requires only three complementary methods—RP-HPLC purity (≥99%), NMR configurational verification, and mass spectrometry—to cover all critical quality attributes of the API [47,52], obviating the need for the complex analytical platforms required by ADCs, such as DAR distribution analysis, free drug residue quantification, and glycan profiling [10]. This disparity is rooted in the chemical nature of PDCs: the single-molecular-entity architecture eliminates the distributive heterogeneity of both conjugation sites and drug loading, streamlining the CMC quality control framework from the “multi-platform, multi-parameter” strategy of ADCs to a “single-component, three-method, full-attribute-coverage” paradigm.

Third, the chemical stability of the amide bond and the chemical lability of the nitrogen mustard moiety—the bis(2-chloroethyl)amino group inherent to the melphalan payload—jointly dictate the “storage” and “administration” design of the formulation [47]. As discussed in Section 3.3, the plasma stability conferred by the amide bond linker provides the chemical basis for intravenous administration, ensuring structural integrity within the circulatory window prior to target cell engagement [47]. Its rapid apparent plasma clearance (α-phase t½ ≈ 1.24 min) is not a signal of chemical instability, but reflects lipophilicity-driven rapid cellular distribution, a distribution pattern that constitutes the pharmacokinetic foundation of Melflufen’s “passive diffusion– enzymatic activation” delivery mechanism [47]. Concurrently, because the nitrogen mustard moiety undergoes hydrolysis in aqueous solution [44], the commercial formulation (Pepaxto^®^) is presented as a lyophilized powder (20 mg/vial), which must be administered within 6 h of reconstitution as a 30 min intravenous infusion [4]. This regimen is delivered on Day 1 of each 28-day cycle in combination with dexamethasone 40 mg; dose reduction to 30 mg is recommended for patients with eGFR 30–45 mL/min/1.73 m^2^ or body weight ≤ 60 kg, to control systemic melphalan exposure [47,53].

Collectively, these CMC attributes—homogeneous DPR, a streamlined analytical framework, and a lyophilized powder presentation—delineate the complete CMC landscape of Melflufen from API synthesis to clinical administration across three dimensions: molecular design, quality control, and formulation development. The central insight of this landscape is that chemical homogeneity itself constitutes an inherent modality advantage of PDCs over ADCs—an advantage that does not depend on any subsequent innovation in linker or payload chemistry, but is rooted in the most fundamental chemical identity of a single-molecular entity. Melflufen’s CMC experience thus provides a reusable translational template for the entire PDC field: the simplicity of the synthetic route defines the lower bound of manufacturing cost, the homogeneity of the chemical structure defines the upper bound of quality control, and the interplay between the chemical stability of the linker and the chemical lability of the payload provides the chemical foundation for the dual design of lyophilized storage and intravenous administration. These principles are by no means unique to Melflufen—they apply to any PDC molecule characterized by a single conjugation site, a fixed drug-to-peptide ratio, and a chemically defined structure, and offer a translational framework and design benchmark for the next-generation PDCs to be discussed in the subsequent sections of this review.

The CMC profile of Melflufen also reflects broader industrialization characteristics intrinsic to the PDC modality. Relative to antibody-based conjugates, peptide-based chemical synthesis affords simpler production and more straightforward scale-up; the chemical homogeneity of PDCs—stemming from their defined drug-to-peptide ratios—translates into more consistent product quality and reduced batch-to-batch variability than the heterogeneous DAR distributions of ADCs; and the overall production cost is lower, owing to chemically defined synthesis and quality control that relies on simpler analytical techniques such as HPLC and mass spectrometry [10]. Lastly, PDCs face persistent regulatory hurdles, as relevant research in this area remains scarce. Strengthening regulatory oversight for PDCs is thus a noteworthy concern.

### 3.5. Comparative Advantages and Clinical Evidence of Melflufen

#### 3.5.1. Preclinical and Translational Evidence

Melflufen has demonstrated substantially enhanced cytotoxic potency compared with its parent alkylator melphalan across multiple preclinical models, with reported increases ranging from approximately 10-fold to several hundred-fold, depending on the experimental system [35]. This variability reflects differences in biological context: in a panel of lymphoma cell lines, melflufen exhibited a mean 49-fold greater potency than melphalan (range 12–102; *p* < 0.001), while in primary patient-derived tumor samples the potency differential was even more pronounced (range 13–455-fold; mean ~108; *p* < 0.001), highlighting marked heterogeneity across experimental conditions and sample sources [54].

At the mechanistic level, Melflufen induces robust DNA damage responses and retains cytotoxic activity irrespective of TP53 status—with comparable induction of apoptosis and cellular stress signaling observed in both TP53-deficient and TP53-proficient multiple myeloma models—suggesting that its cytotoxic action exhibits relatively limited dependence on the canonical p53 pathway [53].This property may hold particular relevance for high-risk disease settings, such as del(17p) multiple myeloma, in which TP53 dysfunction is a defining feature and therapeutic options remain limited.

Collectively, these preclinical findings establish the mechanistic rationale for the clinical evaluation of Melflufen: enhanced intracellular drug delivery, together with a relatively reduced dependence on p53 functional integrity, may jointly contribute to its antitumor activity, although the relative contribution of these mechanisms likely varies across biological contexts.

#### 3.5.2. Clinical Evidence

The transition from preclinical models to the clinic was initially guided by the phase 1/2 O-12-M1 study, which defined a 40 mg intravenous dose administered on Day 1 of a 28-day cycle together with weekly dexamethasone as the recommended regimen for further investigation [18]. The clinical activity of melflufen was further demonstrated in the phase II HORIZON study, which enrolled 157 heavily pretreated patients with RRMM refractory to pomalidomide and/or anti-CD38 monoclonal antibody therapy. The study population had received a median of five prior lines of therapy, with 76% exhibiting triple-class refractory disease and 35% presenting with extramedullary disease. Treatment with melflufen plus dexamethasone achieved an overall response rate (ORR) of 29%, including 26% among triple-class refractory patients. The median duration of response (DOR), progression-free survival (PFS), and overall survival (OS) were 5.5, 4.2, and 11.6 months, respectively. Among responders, median PFS and OS increased to 8.5 and 17.6 months, indicating durable benefit in a subset of patients [5].

The safety profile was characterized primarily by hematologic toxicity, with grade ≥ 3 neutropenia, thrombocytopenia, and anemia occurring in 79%, 76%, and 43% of patients, respectively. Severe non-hematologic adverse events were relatively uncommon, with pneumonia representing the most frequent grade ≥ 3 non-hematologic toxicity (10%). Overall, the results of HORIZON established melflufen plus dexamethasone as an active treatment option for heavily pretreated RRMM, including patients with high-risk clinical and cytogenetic features [5,55].

These findings prompted the randomized phase 3 OCEAN trial (OP-103; NCT03151811), which compared melflufen plus dexamethasone against pomalidomide plus dexamethasone in 495 patients with lenalidomide-refractory disease who had received 2 to 4 prior lines of therapy [27]. The melflufen-containing arm significantly prolonged PFS relative to the control (6.8 vs. 4.9 months; HR = 0.79; *p* = 0.032), yet this improvement did not translate into an OS advantage in the intention-to-treat analysis (HR = 1.10; 95% CI, 0.85–1.44) [46]. Subsequent exploratory work revealed that prior ASCT status strongly modulated the survival outcome: patients who had never undergone ASCT, or whose disease progressed more than 36 months after the procedure, derived the greatest benefit from melflufen, whereas those relapsing within 36 months of ASCT fared worse [41]. On the basis of these data, the EMA authorized melflufen-dexamethasone for adults with triple-class refractory MM who have received three or more prior lines, stipulating a TTP of at least three years in those with a history of ASCT [5,56]. Several subgroup analyses further contextualize the clinical profile of melflufen. Among patients carrying del(17p)—a chromosomal abnormality linked to TP53 inactivation and poor prognosis—the ORR reached 33% with melflufen versus 10.8% with pomalidomide (*p* = 0.028), a differential consistent with the preclinical evidence of p53-independent cytotoxicity [52]. Likewise, a post hoc evaluation restricted to individuals refractory to prior alkylating agents (*n* = 153) found comparable efficacy between the two arms (PFS: 5.6 vs. 4.7 months, HR = 0.92; OS: 23.4 vs. 20.0 months, HR = 0.92), indicating that previous alkylator exposure does not abrogate the therapeutic effect of melflufen [41].

Moving beyond doublet therapy, two triplet regimens were explored. The phase 1/2 ANCHOR study (OP-104) combined melflufen-dexamethasone with either daratumumab or bortezomib, while the phase 3 LIGHTHOUSE trial evaluated melflufen-daratumumab-dexamethasone against daratumumab monotherapy in patients double-refractory to an IMiD and a PI [46]. Both programs were discontinued prematurely for financial reasons rather than for an unfavorable risk-benefit assessment; consequently, the question of whether triplet PDC-based combinations can deliver additive or synergistic clinical value remains unanswered [46].

Taken together, HORIZON and OCEAN collectively establish three clinically actionable conclusions: first, that a mechanism-based PDC can circumvent resistance to PI/IMiD drug classes in a heavily pretreated population; second, that prior ASCT history and alkylator exposure history are the two most informative patient stratification variables for predicting benefit; and third, that del(17p)—a cytogenetic feature associated with p53 dysfunction—may paradoxically enrich for response rather than resistance, a finding that deserves prospective validation in future PDC trials.

#### 3.5.3. Structural Advantages of the PDC Modality over ADC

A side-by-side structural comparison with ADCs—currently the most established class of targeted cytotoxic conjugates—further delineates the differentiating features of Melflufen as a PDC [8]. Belantamab mafodotin, an anti-BCMA antibody–drug conjugate, is a representative ADC for MM treatment. It was approved by the U.S. Food and Drug Administration on October 23, 2025, for clinical use in combination with bortezomib and dexamethasone as the BVd regimen [57,58]. Herein, we analyze the structural differences between PDCs and ADCs from three aspects with belantamab mafodotin as the control.

While the modality-level advantages of PDCs over ADCs have been introduced in Section 2.1, a direct structural comparison with Belantamab mafodotin—a clinically approved anti-BCMA ADC—illustrates how these differences manifest in a disease-relevant context. First, in terms of molecular size, Melflufen (molecular weight 498 Da) and belantamab mafodotin (approximately 152 kDa) differ by approximately 300-fold. The large molecular weight of ADCs restricts their ability to cross biological barriers via passive diffusion, which may limit their capacity to reach tumor cells dispersed throughout the bone marrow compartment [47]. This consideration is particularly relevant in MM, a malignancy characterized by diffuse bone marrow infiltration. Melflufen, by contrast, traverses the plasma membrane via passive diffusion driven by its high lipophilicity, a mechanism that does not require receptor-mediated transcytosis across vascular endothelium (see Section 3.2).

Second, with respect to CMC homogeneity, Melflufen possesses a precisely defined DPR of 1:1 (see Section 3.4), whereas ADCs such as belantamab mafodotin exhibit a heterogeneous DAR distribution (typically DAR = 0–8) [59]. Within this heterogeneous mixture, DAR = 0 species—which lack cytotoxic payload yet retain full antigen-binding capacity—competitively occupy target receptors without contributing to tumor cell killing, while DAR ≥ 4 species undergo accelerated plasma clearance due to increased hydrophobicity, further compressing the effective therapeutic concentration window [6,60,61].

Third, concerning immunogenicity: as a fully synthetic small molecule devoid of immunoglobulin framework regions, Melflufen does not engage Fcγ receptors or activate complement, thereby avoiding the Fc-mediated off-target immune activation that may contribute to the characteristic ocular toxicity profile observed with certain ADCs, including belantamab mafodotin. In the DREAMM-7 trial, ocular adverse events of any grade occurred in 79% of BVd-treated patients (grade ≥ 3, 34%); dose reductions, delays, and discontinuations due to ocular events were required in 44%, 78%, and 9% of patients, respectively [57,58].

It should be emphasized that this comparison is limited to structural and modality-level features and does not constitute an evaluation of the relative clinical efficacy or safety of the two agents. From a drug design platform perspective, however, the single-molecular-entity architecture of Melflufen offers inherent advantages over ADCs across three dimensions—tissue penetration potential, CMC homogeneity, and immunogenicity—that are intrinsic to the PDC modality. The present analysis suggests that PDC-based approaches may offer complementary advantages in specific clinical scenarios, including patients with high bone marrow disease burden or heavily pre-treated disease.

However, these structural advantages must be weighed against the intrinsic pharmacokinetic limitations of the PDC modality. The same small molecular size that confers deep tissue penetration also renders PDCs susceptible to rapid renal filtration—molecules below approximately 25 kDa are cleared within minutes to hours—resulting in a markedly shorter circulatory half-life than the days-to-weeks persistence of antibody-based conjugates [62]. The absence of an Fc domain, while eliminating Fc-mediated off-target toxicity, also forfeits the neonatal Fc receptor (FcRn)-mediated recycling that sustains antibody circulation. In addition, the peptide backbone is vulnerable to serum exo- and endopeptidase degradation, which can further compromise systemic exposure and tumor delivery [62]. These liabilities mean that PDCs and ADCs occupy complementary rather than hierarchically ordered positions: the short half-life and rapid clearance of PDCs reduce the risk of cumulative systemic toxicity but may necessitate more frequent dosing or structural stabilization strategies to maintain therapeutic exposure (discussed further in Section 5.1.3) [41,62], whereas the prolonged circulation of ADCs supports sustained target engagement at the cost of greater cumulative toxicity risk [7]. A balanced appraisal therefore positions the two modalities as offering distinct trade-offs in tissue penetration, circulatory persistence, and toxicity profile, the relative merits of which depend on the specific clinical context.

#### 3.5.4. Non-Overlapping Mechanism of Action Compared with PIs/IMiDs and Clinical Evidence in the Triple-Class-Refractory Setting

At the mechanistic level, the mode of action of Melflufen is distinct from both PIs and IMiDs: it exerts cytotoxicity through DNA crosslinking and the induction of a DNA damage response, whereas PIs primarily target the ubiquitin–proteasome system and IMiDs modulate substrate degradation via cereblon E3 ligase [63]. Because melflufen induces cytotoxicity through DNA crosslinking and activation of the DNA damage response, its mechanism differs fundamentally from those of PIs and IMiDs. This mechanistic distinction may help explain the clinical activity observed in heavily pretreated and triple-class refractory patients [6].

The HORIZON trial tested this concept directly. In this single-arm phase II study, 157 patients with heavily pretreated RRMM received melflufen 40 mg intravenously on Day 1 of each 28-day cycle in combination with weekly dexamethasone (40 mg, or 20 mg for patients aged ≥75 years) [4,5]. As detailed in Section 3.5.2, the doublet achieved clinically meaningful response rates and disease control in a population that was predominantly triple-class refractory (76%), providing clinical proof-of-principle that an alkylating agent-based PDC can circumvent resistance to both proteasome- and cereblon-directed therapies.

At the pharmacodynamic level, preclinical studies provide biological plausibility for the clinical activity observed in HORIZON. Ray et al. demonstrated that Melflufen elicits a rapid DNA damage response, as evidenced by γ-H2AX phosphorylation and activation of downstream ATR/CHK1 signaling within 2 h of exposure in both melphalan-sensitive and melphalan-resistant MM cell lines; by contrast, melphalan induced minimal γ-H2AX in resistant cells even after 24 h of exposure [37]. In addition, melphalan treatment up-regulated the DNA repair protein Ku80, whereas Ku80 induction was not observed following Melflufen exposure, suggesting that Melflufen-induced DNA damage may not fully engage canonical non-homologous end joining (NHEJ) repair [37].

Furthermore, Strese et al. showed that Melflufen exerts anti-angiogenic effects across multiple experimental systems, including reduced endothelial cell viability and inhibition of tube formation in vitro, as well as suppression of angiogenesis in in vivo models where its activity was comparable to that of bevacizumab [64]. These findings suggest that modulation of the tumor microenvironment may also contribute to its therapeutic activity.

Together, these findings provide a plausible biological rationale for the clinical activity of Melflufen in PI/IMiD-refractory settings, although the extent to which these mechanisms translate into clinical benefit remains to be fully defined.

## 4. The Application of PDCs in Different Hematological Malignancies

**Figure 2:**
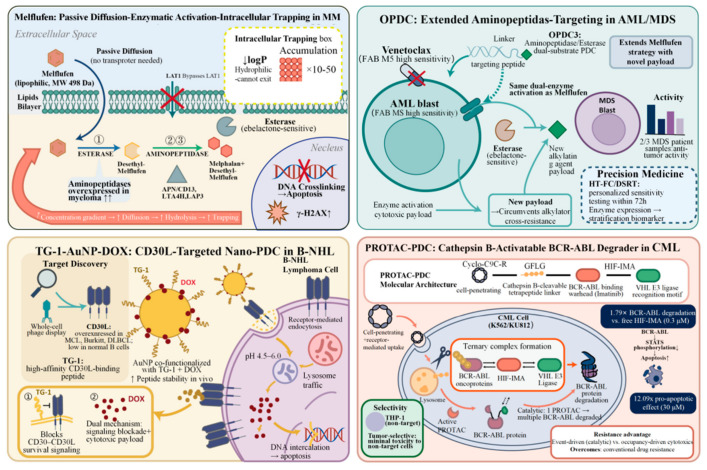
Representative mechanisms of action of peptide–drug conjugates in hematological malignancies. Manually refined based on Refs. [22,28,36,38,65,68].

The following sections examine the application of PDCs across four major hematological malignancy categories, with representative mechanisms of action illustrated in Figure 2.

### 4.1. Extending the Aminopeptidase-Targeting Strategy: OPDC3 in Aml and MDS

OPDC3 shares the aminopeptidase/esterase dual-substrate targeting strategy of Melflufen but incorporates a distinct alkylating payload, offering a therapeutic option for patients whose disease has developed resistance to melphalan-class agents. Preliminary ex vivo functional assessment data have demonstrated that OPDC3 exhibits potent antileukemic activity in patient-derived samples from relapsed/refractory AML [28,65]. Notably, OPDC3 retained robust activity in AML models with acquired resistance to venetoclax, a finding validated both in patient samples and in AML cell lines with acquired venetoclax resistance; moreover, blast cells from FAB M5 subtype patient samples exhibited greater sensitivity to OPDC3 than to venetoclax [65]. Furthermore, OPDC3 demonstrated antitumor activity in MDS blast cells in 4 of 6 patient samples tested [28].

For personalized therapeutic applications, a high-throughput flow cytometry-based drug sensitivity and resistance testing platform (HT-FC/DSRT) can evaluate the responsiveness of primary patient samples to OPDC3 within 72 h. This system lays a methodological framework for selecting tailored PDC candidates guided by ex vivo drug susceptibility data [65]. Notably, the underlying aminopeptidase expression profile is itself heterogeneous across AML FAB subtypes and between individual patients, which may contribute to differential OPDC3 sensitivity [66]. Furthermore, aminopeptidase expression can be modulated by the bone marrow microenvironment, including stromal cell contact and cytokine exposure—variables that are not captured by standard cell-line screening [65]; interpatient variability in enzyme expression may therefore influence therapeutic efficacy. This feature suggests that, in future clinical development, patient stratification strategies based on enzyme expression levels or functional drug sensitivity biomarkers may hold considerable significance.

### 4.2. From Enzyme Substrates to Cell-Surface Neotargets: Discovery and Validation of Cd30L in B-Nhl

The selection of PDC targets is shifting from the classical “known intracellular enzyme” paradigm toward the “de novo discovery of surface targets via phage display.” Shi et al. employed whole-cell phage display to identify the high-affinity peptide TG-1, and subsequent mass spectrometry identified its receptor as CD30L/TNFSF8—a transmembrane ligand protein not previously targeted in B-NHL [22]. Studies have shown that CD30L expression is markedly upregulated across multiple B-NHL subtypes, including mantle cell lymphoma, Burkitt lymphoma, and DLBCL, while its expression in normal peripheral B cells is very low, suggesting its feasibility as a therapeutic target [22]. In contrast to Melflufen, which relies on intracellular enzyme processing, TG-1 specifically binds to CD30L and inhibits lymphoma cell survival and proliferation by blocking the CD30–CD30L signaling axis. To improve in vivo stability and enable combination chemotherapy, the authors further constructed gold nanoparticles (AuNPs) co-functionalized with TG-1 and doxorubicin; the enhanced peptide stability and delivery efficiency conferred by this nano-carrier resulted in highly selective antitumor activity both in vitro and in vivo in B-NHL models [22].

The clinical translation of CD30L-directed PDCs will require standardized detection assays for patient stratification. In the preclinical characterization of TG-1, CD30L expression was assessed at both mRNA and protein levels: mRNA expression was quantified in B-NHL cell lines and primary samples, while cell surface CD30L protein was detected by flow cytometry using a FITC-conjugated anti-human CD30L monoclonal antibody [22]. Notably, no standardized immunohistochemistry (IHC) protocol or scoring system for CD30L has yet been established, in contrast to the well-validated Ber-H2 antibody–based IHC scoring system for CD30 in Hodgkin lymphoma and ALCL. This methodological gap—absence of a clinically validated, IHC-based CD30L detection assay—represents a translational bottleneck for prospective patient selection in CD30L-directed PDC clinical programs [22].

Overall, this study highlights the strategic transition from enzyme-based targeting to cell-surface target discovery and validates the therapeutic potential of CD30L-targeting peptides in B-NHL; however, the clinical translation challenges associated with such nanoparticle-based drugs, including in vivo stability and biodistribution, warrant further investigation.

### 4.3. From Conventional Cytotoxics to Event-Driven Payloads: PROTAC-PDC Targeting BCR-ABL in Hematologic Malignancies

The clinical experiences of Melflufen and OPDC3 have jointly exposed a fundamental limitation: when the payload is a conventional cytotoxin, the efficacy ceiling of a PDC is ultimately constrained by the pre-existing resistance profile of that payload in the target patient population. This recognition has driven a paradigm shift in PDC payload design—from “re-delivering old drugs” toward “event-driven novel payloads,” with proteolysis-targeting chimera-PDCs (PROTAC-PDCs) representing one of the most compelling directions.

In a landmark study, Zhang et al. designed a cathepsin B-activatable covalent PDC-PROTAC specifically tailored to hematologic malignancies. This construct covalently conjugates a leukemia/lymphoma-targeting peptide, Cyclo-C9C-R, via a GFLG tetrapeptide linker to HIF-IMA, a PROTAC molecule that employs imatinib as the target-protein ligand for BCR-ABL and the VHL E3 ligase recognition motif (Leu-Ala-Pro(OH)-Tyr-Ile) as its E3 ligand. This design exploits two features relevant to CML biology: the peptide Cyclo-C9C-R mediates preferential uptake by leukemia and lymphoma cells, while the GFLG linker is selectively cleaved by cathepsin B, a protease overexpressed in multiple hematologic tumor types [36].

In K562 cells (representing BCR-ABL-driven chronic-phase CML), Cyclo-C9C-R-GFLG-HIF-IMA increased the BCR-ABL degradation rate 1.79-fold compared with the unconjugated HIF-IMA control at a concentration as low as 0.3 μmol/L, while simultaneously enhancing the inhibition of downstream STAT5 phosphorylation; at a concentration of 30 μmol/L, the pro-apoptotic effect was 12.09-fold greater than that of unconjugated HIF-IMA. In KU812 cells (representing blast-crisis CML), the same construct induced S-phase cell-cycle arrest in 64.15% of cells at 30 μmol/L and markedly enhanced the inhibition of STAT5 phosphorylation, although its pro-apoptotic effect in KU812 was weaker than that observed in K562, consistent with previous reports that KU812 cells are relatively less sensitive to exogenous stimuli. Importantly, this PDC-PROTAC exhibited negligible cytotoxicity toward THP-1 cells (cell viability consistently >80%), confirming that the “peptide-enzyme-linker” design confers a meaningful degree of tumor selectivity [36].

It should be noted that cathepsin B activity is regulated not only at the expression level, but also by lysosomal pH and endogenous cystatin inhibitors (stefins A/B intracellularly, cystatin C extracellularly) [67]. This regulatory complexity implies that functional protease activity may not be reliably inferred from transcript or protein abundance measurements alone.

Although challenges remain—including inter-individual heterogeneity in enzyme expression, the synthetic complexity arising from the elaborate molecular architecture of these constructs, and the need to evolve from single-trigger toward multi-enzyme/stimulus-responsive linker designs—PROTAC-PDCs represent a pivotal direction for shifting PDC payloads from “occupancy-driven” to “event-driven” pharmacology in hematologic malignancies [36].

The preclinical and clinical evidence for all PDC strategies discussed in this section is comprehensively summarized in Table 1.

## 5. Challenges, Limitations, and Future Perspectives

### 5.1. Current Challenges and Limitations of PDC Development

#### 5.1.1. Antigen Expression Heterogeneity and Patient Selection

Across all PDC strategies reviewed in Section 4—from the aminopeptidase-targeted melflufen and OPDC3 to the CD30L-directed TG-1 conjugate—therapeutic efficacy is fundamentally contingent upon sufficient expression of the molecular target within the tumor cell population. For example, Aminopeptidase expression varies substantially across AML subtypes and between individual patients, and CD30L expression in B-NHL is subtype-dependent, being most consistently high in mantle cell lymphoma and Burkitt lymphoma while showing more variable levels in DLBCL [22,37,53]. Current PDC research in hematological malignancies is concentrated in MM and AML, with emerging preclinical and early clinical data extending into B-NHL(CD30L-directed TG-1 conjugate, Section 4.2), chronic myeloid leukemia (BCR-ABL-targeted PROTAC-PDC, Section 4.3), and MDS (aminopeptidase-targeted OPDC3, Section 4.1). By contrast, CLL remains at an early exploratory stage without key data to guide clinical development [69], and concept-validation evidence for ALL-directed PDCs is even more limited. Fundamental questions persist: what constitutes the minimum receptor density threshold for adequate PDC intracellular delivery? Should systematic target expression profiling be incorporated as an eligibility criterion in future PDC clinical trials?

#### 5.1.2. Intrinsic Payload Resistance and Strategic Limitations

The OCEAN trial experience carries generalizable lessons beyond melflufen itself: prior high-dose melphalan (HDM) exposure is a negative predictive factor for alkylating agent-class PDCs. As detailed in Section 3.5.2, the alkylator-refractory subgroup of OCEAN demonstrated comparable outcomes between the two treatment arms, indicating that enhanced intracellular delivery alone cannot overcome pre-established resistance to the payload class. This finding carries a generalizable implication: the therapeutic ceiling of any PDC is ultimately constrained by the intrinsic resistance profile of its payload in the intended patient population, irrespective of how efficiently the conjugate achieves tumor-selective delivery. OPDC3 addresses this by substituting a non-melphalan payload, partially circumventing alkylator cross-resistance. PROTAC-PDCs face a distinct resistance concern: downregulation or mutation of the E3 ubiquitin ligase subunit (e.g., VHL) could render the ternary complex non-functional, representing a mechanism of acquired resistance not encountered with conventional cytotoxic payloads [36]. Dual-payload PDC designs and rational combination strategies with PDC remain experimentally unvalidated in hematological malignancies. Beyond payload resistance, the clinical development of melflufen also provides broader lessons for future PDC design. First, tumor selectivity can be achieved through the integration of linker chemistry with tumor-specific biology, as exemplified by the passive-diffusion–enzymatic-activation–intracellular-trapping cascade underlying melflufen. Second, the OCEAN experience demonstrated that biomarker-informed patient selection—including prior ASCT history and treatment-free interval—may be a stronger determinant of clinical benefit than disease classification alone. Together, these observations highlight the importance of mechanism-driven design and prospective patient stratification in future PDC development.

#### 5.1.3. Pharmacokinetic Limitations and Circulatory Stability

The intrinsic liabilities of peptide-based therapeutics—susceptibility to serum endo- and exopeptidase-mediated degradation and rapid renal filtration of molecules below approximately 25 kDa—constrain tumor exposure of PDCs and limit their therapeutic window [62]. Structural strategies to extend plasma half-life include backbone cyclization, incorporation of D-amino acids or non-natural amino acid analogues, PEGylation, albumin-binding peptide tags, and encapsulation within high-molecular-weight polymeric carriers such as HPMA copolymers, each of which must be balanced against potential compromise of tumor penetration capacity [41,62]. A translational consideration specific to enzyme-activated PDCs is the interspecies difference in plasma carboxylesterase (CE) activity: murine plasma CE activity is approximately 10-fold higher than in human plasma, meaning that Val-Cit-containing or ester-based PDCs may undergo substantially faster non-specific cleavage in rodent pharmacokinetic models than would be expected clinically [11]. Taken together, these fundamental pharmacokinetic challenges underscore a central tension in PDC development: the design features that enable rapid tumor penetration and clearance (small size, lack of Fc region) are the very same features that limit circulatory residence time and tumor drug exposure. These pharmacokinetic hurdles are not insurmountable, but their resolution demands that linker stability, peptide backbone engineering, and species-appropriate preclinical models be addressed in an integrated rather than piecemeal fashion—a challenge that the PDC field has only begun to tackle systematically.

#### 5.1.4. Insufficient Maturity of Clinical Evidence

As summarized in Table 1, the clinical evidence base for PDCs in hematological malignancies remains highly heterogeneous across disease subtypes. From a preclinical standpoint, the available data derive predominantly from established cell lines and immunodeficient xenograft models, which do not adequately recapitulate the bone marrow microenvironmental factors—stromal adhesion, cytokine networks, hypoxia, and drug transporter upregulation—that contribute to treatment resistance in patients. The CD30L-directed TG-1 conjugate and the BCR-ABL-targeted PROTAC-PDC strategies remain at the preclinical stage, and OPDC3 efficacy data are confined to ASH conference abstracts without peer-reviewed publication [22,28,37]. Translational extrapolation from these platforms is further complicated by the absence of validated biomarkers or standardized preclinical models that can reliably predict clinical PDC activity across hematological disease subtypes.

On the clinical side, the evidence base is equally fragile. Melflufen remains the only PDC to have completed a phase III randomized trial in a hematological malignancy; although it demonstrated superior PFS versus pomalidomide in the OCEAN trial, it failed to achieve a statistically significant overall survival benefit in the intent-to-treat population, leading to FDA withdrawal in 2024 [70]. No other PDC has progressed beyond phase II evaluation in this disease area—OPDC3 and PROTAC-PDC platforms remain in preclinical or early-stage development—and head-to-head comparative data between PDC-based approaches and current standard-of-care modalities, including ADCs, bispecific T-cell engagers, and CAR-T therapies, are entirely absent. From a regulatory perspective, neither the FDA nor the EMA has issued PDC-specific development guidance; regulatory evaluation currently relies on frameworks adapted from ADC or small-molecule drug pathways, which may inadequately capture the distinctive pharmacokinetic and pharmacodynamic properties of this modality. To better contextualize the position of PDCs among currently available targeted and immune-based therapeutic platforms, we summarized the major differences between PDCs, ADCs, bispecific antibodies, and CAR-T-cell therapies in terms of molecular size, manufacturing complexity, toxicity profile, tumor penetration, cost, and clinical maturity (Table 2) [6,7,12,13,25,48,49,71,72,73,74,75,76]. Overall, PDCs offer potential advantages in small molecular size, chemical manufacturability, and tissue penetration; however, their clinical evidence base remains substantially less mature than that of ADCs, bispecific antibodies, and CAR-T-cell therapies. In contrast, ADCs and T-cell–redirecting therapies have already achieved multiple regulatory approvals and have been incorporated into treatment algorithms for several hematological malignancies, although they are associated with distinct challenges, including complex biologic manufacturing, high cost, antigen-dependent resistance, and modality-specific toxicities. Addressing these limitations will require biomarker-guided patient selection, more predictive disease models, and regulatory frameworks tailored to the unique pharmacological characteristics of peptide-based conjugates.

### 5.2. Future Perspectives

#### 5.2.1. Linker Chemistry and Payload Innovation

Linker engineering is evolving from conventional single-stimulus-responsive systems toward increasingly sophisticated architectures capable of integrating multiple tumor-associated cues, including enzymatic activity, acidic pH, and redox conditions. Such multi-trigger designs have the potential to further improve tumor selectivity while minimizing premature payload release. In parallel, payload pharmacology is undergoing a transition from occupancy-driven cytotoxic agents toward event-driven therapeutic modalities. As demonstrated by Zhang et al. (Section 4.3), cathepsin B-activatable PROTAC-PDCs achieve catalytic target degradation rather than stoichiometric cytotoxicity—a pharmacological distinction with direct implications for overcoming payload resistance [36]. Looking forward, the extension of this event-driven framework to other oncogenic drivers beyond BCR-ABL, combined with multi-stimulus linker designs capable of restricting PROTAC release to the lysosomal compartment, represents a priority direction for next-generation PDC payload development.

#### 5.2.2. Nanotechnology-Enhanced PDC Delivery

Beyond linker and payload optimization, nanotechnology-enabled delivery systems provide complementary approaches to address the intrinsic pharmacokinetic limitations of peptide-based therapeutics. At the inorganic nanocarrier frontier, TG-1 and doxorubicin co-functionalized gold nanoparticles exert highly selective antitumor activity in in vitro and in vivo models of B-NHL [22]. An alternative, polymer-based approach employs high-molecular-weight carriers such as HPMA copolymers to circumvent renal filtration while exploiting EPR-mediated tumor accumulation. Li et al. recently reviewed HPMA copolymers as a versatile platform for peptide-drug delivery [62]. In the hematological context, the value of such macromolecular carriers derives from mechanisms that operate independently of passive tumor accumulation. First, the high molecular weight of HPMA-peptide conjugates exceeds the glomerular filtration threshold, circumventing the rapid renal clearance that otherwise limits the systemic exposure of small peptides and thereby substantially prolonging their circulation time. Second, the HPMA scaffold functions as a “molecular shield” that protects conjugated peptides from enzymatic degradation, while simultaneously enabling the multifunctional conjugation of tumor-targeting ligands, stimulus-responsive cleavable linkers, and imaging probes within a single construct—supporting receptor-mediated active targeting and theranostic applications. As these mechanisms do not depend on the EPR effect, they remain applicable to circulating and bone marrow-resident tumor cells in hematological malignancies [62].

#### 5.2.3. Biomarker-Guided Precision PDC Therapy

The post hoc experience from the OCEAN trial (discussed in Section 3.5.2 and Section 5.1.2) demonstrates that histology-based enrollment criteria are insufficient for PDC clinical programs, and that outcome heterogeneity can only be retrospectively explained—rather than prospectively prevented—without integrated biomarker stratification. These findings converge on a unified lesson—future clinical programs should integrate multidimensional eligibility criteria encompassing target enzyme expression levels, tumor mutational landscape, and prior treatment exposure history, rather than relying on histology-based enrollment alone [41,43].

#### 5.2.4. Artificial Intelligence-Assisted PDC Discovery and Development

Artificial Intelligence and deep learning are beginning to transform the rational design pipeline for PDC components. At the level of targeting peptide discovery, recent generative protein design frameworks such as RFdiffusion enable the de novo creation of peptide binders with predefined structural constraints, thereby expanding the accessible sequence space beyond that achievable through conventional phage display or combinatorial screening approaches [42]. Complementing de novo peptide generation, deep learning-based protein sequence design frameworks such as ProteinMPNN have demonstrated the ability to redesign amino acid sequences while preserving desired structural features and biological functions [77]. Although originally developed for protein engineering, these advances highlight the potential of AI-guided sequence optimization strategies to improve the stability, binding properties, and developability of peptide ligands, which may ultimately facilitate the rational design of next-generation PDCs. Advances in structure-based design further enhance this process. Protein structure prediction platforms such as AlphaFold2 allow high-resolution modeling of target proteins and peptide–receptor interaction interfaces, thereby supporting rational optimization of peptide binding and target selectivity [78]. Moving from peptide design to linker engineering, reinforcement learning platforms such as DRlinker enable the targeted generation of linker candidates with defined chain lengths and lipophilicity, achieving 91% compliance with specified linker length constraints and 94% compliance with desired logP ranges while maintaining predicted bioactivity [79]. These computational approaches may be particularly valuable for optimizing enzyme-cleavable linkers in PDCs, where linker stability and payload-release kinetics critically influence therapeutic efficacy and safety.

Beyond molecular design, machine-learning approaches are increasingly being applied across multiple stages of drug discovery, including target identification, biomarker discovery, and candidate prioritization through the integration of transcriptomic, proteomic, and single-cell sequencing datasets [80]. In parallel, AI-assisted pharmacokinetic and ADMET prediction models are emerging as valuable tools for estimating peptide stability, tissue distribution, systemic clearance, and toxicity profiles during early-stage drug development, thereby improving candidate prioritization before experimental validation [81].

Underpinning these computational advances, dedicated databases such as PDCdb compile standardized biological activity and pharmaceutical information from clinical studies, animal models, and cell-based assays, providing the curated training resources essential for AI-driven model development [78]. As algorithmic sophistication, structural biology resources, and high-quality datasets continue to expand, AI-assisted PDC development is expected to accelerate peptide optimization, linker selection, target identification, and pharmacokinetic profiling, ultimately reducing development timelines and preclinical attrition rates.

## 6. Conclusions

Peptide–drug conjugates (PDCs) represent an emerging class of targeted therapeutics that combine the tumor-targeting capabilities of peptides with the cytotoxic potency of small-molecule payloads. By integrating advances in peptide engineering, linker chemistry, and payload design, PDCs offer a versatile platform capable of addressing several limitations associated with conventional chemotherapy and other targeted drug delivery systems.

Although clinical experience remains limited, the studies completed to date have demonstrated the feasibility of achieving selective drug delivery and meaningful antitumor activity in hematological malignancies. At the same time, challenges related to payload resistance, patient stratification, clinical validation, and regulatory development highlight that the field is still in an early stage of maturation.

Future progress will likely depend on the convergence of multiple innovations, including biomarker-guided patient selection, multi-stimulus-responsive linker systems, event-driven payloads such as PROTACs, nanotechnology-enabled delivery strategies, and artificial intelligence-assisted molecular design. As these technologies continue to evolve and more clinical evidence becomes available, PDCs have the potential to become an important component of precision therapeutic strategies for hematological malignancies.

## Figures and Tables

**Table 1:** Summary of Peptide–Drug Conjugates in Hematological Malignancies.

PDC Name	Target	Payload	Linker	Indication	Preclinical Key Finding	Development Status	Key Efficacy	Key Safety (Grade ≥ 3)	References
MULTIPLE MYELOMA									
Melflufen (Melphalan flufenamide) Pepaxto	Aminopeptidases (APN/CD13, LTA4H, LAP3) Enzyme substrate type	Melphalan (Nitrogen mustard DNA alkylator	Amide bond Esterase then Aminopeptidase sequential cleavage	RRMM	C50 12-455-fold > Mel (mean ~108) 10–50× intracellular Mel accumulation TP53-independent cytotoxicity Active in Mel-resistant cell lines [30,38,41]	FDA: Withdrawn in 2024 EMA/MHRA: Conditional approval maintained	ORR 29% mDOR 5.5 mo, mPFS 4.2 mo OCEAN (Ph3, *n* = 495): mPFS 6.8 vs. 4.9 mo (HR 0.79) del(17p): ORR 33% vs. 10.8%	Neutropenia 79% Thrombocytopenia 76% Anemia 43% Pneumonia 10% Non-heme AE: uncommon	[4,5,24,30,39,41,57]
AML/MDS									
OPDC3	Aminopeptidases/Esterases Enzyme substrate type	Proprietary alkylating agent (DNA crosslinking; structure undisclosed)	Amide bond (Esterase + Aminopeptidase sequential cleavage)	AML MDS	Active in venetoclax-resistant AML patient samples and cell lines FAB M5 blasts: more sensitive to OPDC3 than venetoclax Activity in 4/6 MDS samples [28,65]	Preclinical	Active in AML samples and venetoclax-resistant AML models.	Unknown (no human safety data)	[28,65]
B-NHL (MCL, BL, DLBCL)									
TG-1 Peptide Conjugate (AuNP-TG-1-Dox)	CD30L/TNFSF8 Receptor-targeted (phage display discovery)	Doxorubicin (Anthracycline Topo II inhibitor)	Gold nanoparticle (AuNP) carrier platform (non-classical linker)	MCL, BL, DLBCL	CD30L high in B-NHL, low in normal B cells Dual mechanism: Dox cytotoxicity + CD30-CD30L signaling blockade Selective killing in vitro and in vivo [20]	Preclinical	Selective anti-lymphoma activity in CD30L-positive B-NHL models.	Favorable window (CD30L low in normal tissue)	[22]
CML									
PROTAC-PDC (Cyclo-C9C-R-GFLG- HIF-IMA)	BCR-ABL/Leukemia cells via Cyclo-C9C-R cell-penetrating peptide	HIF-IMA (PROTAC) Imatinib ligand + VHL E3 ligase ligand	GFLG tetrapeptide Cathepsin B-cleavable	CML (Chronic & blast crisis)	- K562 (CP-CML): BCR-ABL degradation 1.79× at 0.3 uM Apoptosis 12.09× at 30 uM vs. free PROTAC - KU812 (BC-CML): S-phase arrest 64.15% - THP-1 viability > 80% (selectivity confirmed) - Also validated in U87MG glioma [29,58]	Preclinical	Cell line data only (K562, KU812) Enhanced BCR-ABL degradation and anti-leukemic activity in CML cell models.	Low cytotoxicity to THP-1 (>80% viability) Event-driven mechanism	[29,58]

Abbreviations: RRMM, relapsed/refractory multiple myeloma; AML, acute myeloid leukemia; MDS, myelodysplastic syndromes; B-NHL, B-cell non-Hodgkin lymphoma; MCL, mantle cell lymphoma; BL, Burkitt lymphoma; DLBCL, diffuse large B-cell lymphoma; CML, chronic myeloid leukemia; CP-CML, chronic phase CML; BC-CML, blast crisis CML; ORR, overall response rate; mDOR, median duration of response; mPFS, median progression-free survival; AE, adverse event; APN/CD13, Aminopeptidase N; BCR-ABL, breakpoint cluster region-Abelson; GFLG, Gly-Phe-Leu-Gly tetrapeptide; HIF-IMA, hypoxia-inducible factor–imatinib conjugate; PROTAC, proteolysis targeting chimera; TG-1, phage display-derived CD30L-targeting peptide; TNFSF8, TNF superfamily member 8; VHL, Von Hippel-Lindau E3 ubiquitin ligase.

**Table 2:** Comparative features of PDCs, ADCs, bispecific antibodies, and CAR-T therapies in hematological malignancies.

Features	PDCs	ADCs	Bispecific Antibodies	CAR-T Therapies
Molecular size	~0.5 kDa	~150 kDa	~55; ~146–150; ~197	Not applicable as molecular weight. Cell diameter: approximately 7–10 μm for individual T cells
Manufacturing complexity	Low to moderate. Chemical synthesis ensures homogeneous products, and single-site conjugation enables a fixed drug-to-peptide ratio and simplified quality control.	High. Require recombinant antibody production, linker–payload conjugation, purification, DAR control, and extensive analytical characterization	Moderate to high. Format-specific engineering and purification are required to ensure correct chain pairing, heterodimer formation, aggregation control, and removal of mispaired byproducts.	Very high. Patient-specific living cellular products; manufacturing typically involves leukapheresis, T-cell isolation/activation, genetic modification, ex vivo expansion, formulation, cryopreservation, release testing, and individualized logistics.
Toxicity profile	Generally lower immunogenicity; mainly characterized by hematologic toxicities such as neutropenia, thrombocytopenia, and anemia.	Peripheral neuropathy and ocular toxicity for selected ADCs.	CRS, ICANS, infections, cytopenias, and hypogammaglobulinemia.	CRS, ICANS, prolonged cytopenias, infections, hypogammaglobulinemia/B-cell aplasia, and rare secondary malignancies.
Tumor penetration	Favorable tissue and cellular penetration, high lipophilicity-driven passive diffusion; rapid renal clearance may limit systemic exposure.	Limited by large antibody-based molecular size; tumor-site access depends on vascular access, antigen binding, internalization, and payload release.	Format-dependent; small BiTE formats may distribute more readily but have short half-lives, whereas IgG-like bispecific antibodies have longer systemic exposure and antibody-like tissue distribution.	Not governed by molecular diffusion; efficacy depends on CAR-T-cell expansion, persistence, antigen recognition, and access to circulating, bone marrow–resident, lymphoid, or extramedullary malignant cells.
Cost	Relatively low.	High.	High.	Very high.
Clinical maturity	Early stage. Only one PDC has completed a phase III randomized trial in a hematological malignancy.	High. Multiple approved agents.	Moderate to high. Rapidly expanding regulatory approvals.	High. Multiple approved CAR-T products targeting CD19 or BCMA.

PDCs, peptide–drug conjugates; ADCs, antibody–drug conjugates; CAR-T, chimeric antigen receptor T; DAR, drug-to-antibody ratio; CRS, cytokine release syndrome; ICANS, immune effector cell-associated neurotoxicity syndrome; BiTE, bispecific T-cell engager; BCMA, B-cell maturation antigen.

## Data Availability

No new data were created or analyzed in this study.
